# Modulation of Cardiac Alternans by Altered Sarcoplasmic Reticulum Calcium Release: A Simulation Study

**DOI:** 10.3389/fphys.2018.01306

**Published:** 2018-09-19

**Authors:** Jakub Tomek, Markéta Tomková, Xin Zhou, Gil Bub, Blanca Rodriguez

**Affiliations:** ^1^Department of Computer Science, British Heart Foundation Centre of Research Excellence, University of Oxford, Oxford, United Kingdom; ^2^Nuffield Department of Medicine, University of Oxford, Oxford, United Kingdom; ^3^Department of Physiology, McGill University, Montreal, QC, Canada

**Keywords:** alternans, calcium handling, calcium release, calcium release dynamics, sarcoplasmic reticulum cycling, computer modeling

## Abstract

**Background:** Cardiac alternans is an important precursor to arrhythmia, facilitating formation of conduction block, and re-entry. Diseased hearts were observed to be particularly vulnerable to alternans, mainly in heart failure or after myocardial infarction. Alternans is typically linked to oscillation of calcium cycling, particularly in the sarcoplasmic reticulum (SR). While the role of SR calcium reuptake in alternans is well established, the role of altered calcium release by ryanodine receptors has not yet been studied extensively. At the same time, there is strong evidence that calcium release is abnormal in heart failure and other heart diseases, suggesting that these changes might play a pro-alternans role.

**Aims:** To demonstrate how changes to intracellular calcium release dynamics and magnitude affect alternans vulnerability.

**Methods:** We used the state-of-the-art Heijman–Rudy and O’Hara–Rudy computer models of ventricular myocyte, given their detailed representation of calcium handling and their previous utility in alternans research. We modified the models to obtain precise control over SR release dynamics and magnitude, allowing for the evaluation of these properties in alternans formation and suppression.

**Results:** Shorter time to peak SR release and shorter release duration decrease alternans vulnerability by improved refilling of releasable calcium within junctional SR; conversely, slow release promotes alternans. Modulating the total amount of calcium released, we show that sufficiently increased calcium release may surprisingly prevent alternans via a mechanism linked to the functional depletion of junctional SR during release. We show that this mechanism underlies differences between “eye-type” and “fork-type” alternans, which were observed in human *in vivo* and *in silico*. We also provide a detailed explanation of alternans formation in the given computer models, termed “sarcoplasmic reticulum calcium cycling refractoriness.” The mechanism relies on the steep SR load–release relationship, combined with relatively limited rate of junctional SR refilling.

**Conclusion:** Both altered dynamics and magnitude of SR calcium release modulate alternans vulnerability. In particular, slow dynamics of SR release, such as those observed in heart failure, promote alternans. Therefore, acceleration of intracellular calcium release, e.g., via synchronization of calcium sparks, may inhibit alternans in failing hearts and reduce arrhythmia occurrence.

## Introduction

Repolarization alternans, the alternation of long and short action potential durations (APD), has been linked to the incidence of ventricular fibrillation and sudden cardiac death (Pastore et al., 1999; Wilson and Rosenbaum, 2007). Episodes of fibrillation were observed to be consistently preceded by alternans formation both in ventricles (Wilson et al., 2009) and atria (Narayan et al., 2011), suggesting that alternans importantly contributes to arrhythmogenesis. At the cellular level, repolarization alternans has been extensively researched both experimentally and computationally (Díaz et al., 2004; Szentesi et al., 2004; Weiss et al., 2006; Livshitz and Rudy, 2007; Kanaporis and Blatter, 2015; Bayer et al., 2016; Qu et al., 2016; Zhou et al., 2016; Tomek et al., 2017). Alternans may be driven primarily by steep APD restitution, or by oscillations in calcium handling (Pruvot et al., 2004). In the calcium-driven alternans hypothesis, calcium alternans consist of calcium transient amplitude oscillation between subsequent beats, which then translate into repolarization alternans via sodium-calcium exchanger and other calcium-sensitive currents (Livshitz and Rudy, 2007; Edwards and Blatter, 2014).

Multiple heart diseases are associated with an increased vulnerability to alternans, particularly heart failure (Kodama et al., 2004; Wilson et al., 2009), as well as hypertrophic cardiomyopathy (Cannon et al., 1986), or myocardial infarction (Gardner et al., 2015). It is known that cardiac remodeling in heart failure and other diseases alters both intracellular calcium release (Lindner et al., 2002) and reuptake (Kho et al., 2012) within cardiomyocytes. While reduced reuptake capacity has been linked to alternans vulnerability (Weiss et al., 2006; Qu et al., 2007), the relationship between altered calcium release and alternans vulnerability is less clear. Ryanodine receptor leakiness has been identified both as a promotor and a suppressor of alternans (Lehnart et al., 2006; Xie et al., 2008). However, the precise mechanisms by which calcium release properties regulate alternans vulnerability in large mammalian hearts are not known. This may be important to better understand pro-arrhythmia mechanisms in diseased hearts with altered calcium release mechanisms.

The main goal of this work is to characterize how altered properties of the sarcoplasmic reticulum (SR) calcium release modulate alternans vulnerability, contributing to the explanation of alternans vulnerability in heart failure and other diseases. We focus at two features of SR release: the dynamics (how quickly is a given amount of calcium released) and magnitude (how much calcium is released in total), showing that both factors modulate alternans. To study the properties of altered calcium release with respect to alternans, we utilize computational modeling of cardiomycytes, which provides excellent observability and control of the studied systems. We used the state-of-the-art model of ventricular myocyte by Heijman et al. (2011), which is the most up-to-date version of the Hund–Rudy model (Hund and Rudy, 2004; Livshitz and Rudy, 2007; Decker et al., 2009) and the ORd model (O’Hara et al., 2011). Both models have been extensively validated against experimental data, contain a detailed model of calcium handling, and were used for study of various aspects of alternans (Livshitz and Rudy, 2007; Zhou et al., 2016; Tomek et al., 2017). The major advantage of these models is the compartmentalization of the SR. This allows the study of calcium cycling within SR, which was suggested to be an important modulator of cellular calcium handling (Ramay et al., 2011) and alternans (Kihara and Morgan, 1991). We demonstrate in this work that this cycling underlies alternans in the studied models and that this is important for the interpretation of our results on alternans and SR release properties.

## Materials and Methods

### Computational Models of Membrane Kinetics and Calcium Handling

**FIGURE 1:**
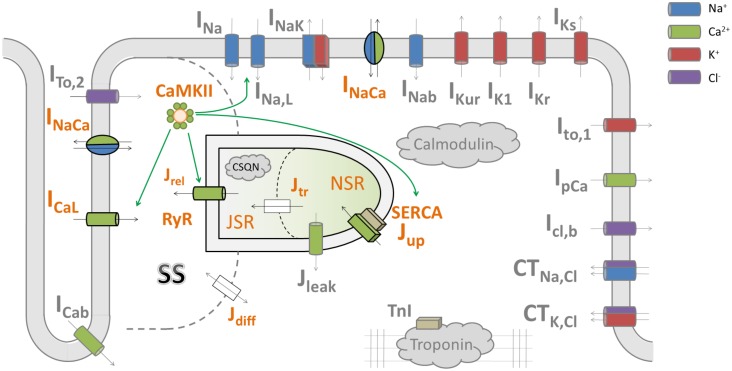
A diagram of the HeRd model of ventricular cardiomyocyte and an overview of calcium handling. Shown are transmembrane currents, compartmentalized sarcoplasmic reticulum and the associated fluxes, and CaMKII signaling. β-adrenergic signaling components in the model were not used in this study. The calcium handling components relevant for alternans are highlighted in orange font. Green arrows indicate which components are regulated by CaMKII, an important regulator of calcium handling. Within an action potential, calcium handling in the model works as follows: L-type calcium current (I_CaL_) induces an influx of calcium which is sensed by ryanodine receptors (RyR). This induces a large-magnitude release of calcium, J_rel_, via RyR (this process is termed CICR: calcium-induced calcium release). The amount of calcium released via RyR depends mainly on the calcium stimulus via I_CaL_ and the load–release relationship of junctional sarcoplasmic reticulum containing RyR (JSR): the fuller the JSR is, the more calcium it releases (Shannon et al., 2000). This relationship is steep, as shown in a previous study of the Hund-Rudy model (Livshitz and Rudy, 2007) and experiments (Shannon et al., 2000), i.e., a relatively small increase in JSR contents may increase fractional release considerably. The released calcium then diffuses to the intracellular space (J_diff_) and eventually to SERCA pumps, which reuptake the calcium back to network sarcoplasmic reticulum containing SERCA pumps (NSR). Ultimately, within sarcoplasmic reticulum, calcium diffuses from NSR to JSR along the concentration gradient (J_tr_), from where it may be released in the next action potential. SS codes the junctional calcium subspace where the calcium influx via ICaL stimulates ryanodine receptors. The figure is based on Heijman et al. (2011).

The primary model used in this study is Heijman–Rudy (HeRd) (**Figure 1**), a model of canine ventricular myocyte (Heijman et al., 2011); the β-adrenergic component of the model was not used in this study. In addition, we used the O’Hara–Rudy (ORd) model of human ventricular myocyte (O’Hara et al., 2011) in Section Results: Increased and Decreased Magnitude of SR Release Attenuates Alternans to directly link our mechanistic insight to a previously published study utilizing this model. The ORd model has the same compartmentalization as HeRd, but contains human-specific formulations of ionic currents. Codes for both models were downloaded from the website of the lab of Prof. Yoram Rudy^1^.

Action potential duration (APD) alternans at a given time point was defined as the difference in APD90 (APD at 90% repolarization level) between two consecutive action potentials.

Below is listed methodology for the respectively numbered “Results” sections.

### Stimulation Protocols

At all pacing rates, simulated cells were paced for 2500 beats to reach quasi-stable state as shown previously (Tomek et al., 2017). In a system manifesting alternans, true stability is impossible, given the presence of oscillations in APD and ionic concentrations. We term the cellular model to be in a quasi-stable state when the maximum and minimum of each of the following variables during two consecutive beats change less than 0.2% per 100 beats: membrane potential, intracellular calcium, intracellular potassium, and intracellular sodium. Maxima and minima of beat pairs are used to allow cells manifesting alternans to be considered stabilized. For convenience, “stable state” is used instead of “quasi-stable state” in the rest of the article.

When evaluating the effect of altered time constant of diffusion between the network sarcoplasmic reticulum (NSR) and the junctional sarcoplasmic reticulum (JSR), cells were first pre-paced for 2500 beats at base cycle lengths 260, 300, and 340. Subsequently, the pre-paced cells were loaded and paced for 2500 more beats with both reduced and increased time constant (60, 70,...,140% of original value) and alternans was measured at the end of the 2500 beats.

### Model Modifications to Investigate SR Release Dynamics

**FIGURE 2:**
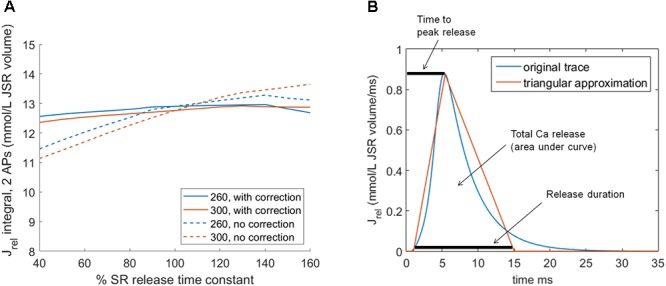
Altering SR release dynamics at bcl 260 and 300 in the HeRd model: **(A)** The relationship between altered time constant of RyR release and total amount of calcium released over two consecutive action potentials at two pacing rates. In solid lines is shown the relationship after the application of compensatory rescaling of I_CaL_ conductance, as described in the main manuscript. The dashed lines show how the relationship between altered SR release time constant and total calcium release would have looked without such scaling. **(B)** An illustration of the approximation of native HeRd J_rel_ using a triangle. Throughout the article, parameters varied are the release duration, time to peak release, and total calcium released.

To modulate the key features of SR release dynamics, the time to peak release and release duration, we scaled the ryanodine receptor (RyR) release time constant. While changing the RyR release time constant exerts only a small effect on total amount of calcium released (lowering time constant slightly lowers total calcium released and vice versa), it could nevertheless theoretically confound the result achieved. To account for this, we scaled the L-type calcium current (I_CaL_) conductance in addition to the time constant to achieve a near–flat relationship between the time constant and total calcium released (**Figure 2A**, solid lines; dashed lines show the relationship without I_CaL_ scaling). For time constant multipliers of 0.4, 0.5,...,1.6, the corresponding multipliers of I_CaL_ conductance at base cycle length (bcl) 260 were: 1.0823, 1.0643, 1.0479, 1.0334, 1.0204, 1.0057, 1.0000, 0.9949, 0.9901, 0.9854, 0.9810, 0.9673, and 0.9545. At bcl 300, the corresponding I_CaL_ conductance multipliers were: 1.0949, 1.0772, 1.0593, 1.0425, 1.0269, 1.0129, 1.0000, 0.9882, 0.9772, 0.9690, 0.9650, 0.9605, and 0.9558.

In order to separate two aspects of SR release dynamics, the time to peak release and the release duration, we modified the model to allow a clamp of SR release J_rel_ in the form of a triangle approximating a native release (**Figure 2B**). Concretely, the triangular release starts 1 ms after the stimulus administration (similarly to normal SR release) and has three parameters: release duration (base width of the triangle), time to peak (position of the triangle peak), and total calcium released (area of the triangle).

The triangular clamp was applied after a cell was pre-paced to a stable state using its native SR release formulation. The prepacing was performed for 2500 beats at 400 ms bcl. At this pacing rate the cell does not manifest alternans, but the pacing frequency is fast enough to activate CaMKII and other rate-dependent mechanisms. Calcium leak from JSR (I_leak,JSR_) was set to 0 when the triangular clamp was used so that the total SR release was equal to the value imposed by the triangular clamp and it was not “contaminated” by additional JSR leak.

In order to assess the vulnerability of cells with clamped SR release to alternans, we use a proxy termed “alternans threshold” related to the capability of the cell to refill the JSR following a calcium release. Specifically, we loaded from a file a pre-paced stable-state cell and simulated a single AP with a clamped SR release of given parameters, observing the ability of the cell to refill JSR to the same value as at the beginning of the AP within 400 ms. We searched a range of values of release duration (5–40 ms) and time to peak release (2–35 ms).

• For each of these combinations, we searched a range of values of the total amount of calcium released (effectively stretching the release triangle vertically). Each tested combination of release duration, time to peak release, and total amount of calcium released was simulated after loading a pre-paced stable state, i.e., the simulations were entirely independent.
• “Alternans threshold” is the level of total calcium released above which a cell with the given release duration and time to peak release fails to refill the JSR to the pre-release state within the APD. The intuition underlying the term “alternans threshold” is that in the given family of models, alternans starts following a failure to refill the JSR, which then leads to further oscillation of the calcium handling subsystem.

### Modulating SR Release Magnitude

**FIGURE 3:**
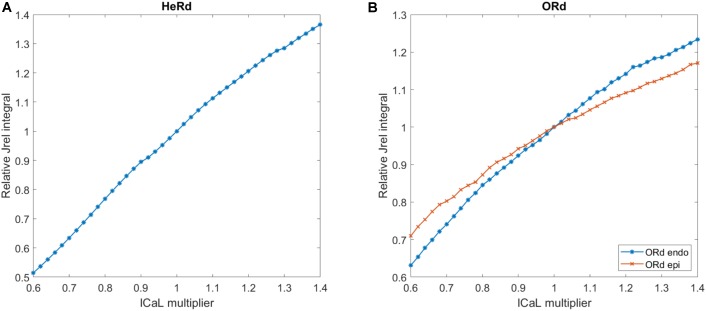
Near-linear relationship between I_CaL_ multiplier and total SR release in the HeRd and ORd models. Shown are results in the HeRd model **(A)** and the ORd model, endocardial, and epicardial **(B)**. Relative increase in total SR release (J_rel_ integral) for a given I_CaL_ scaling factor is given as follows: let *x* be an I_CaL_ scaling factor and *y* be the resulting integral of J_rel_ over two action potentials after stable-state pre-pacing. Let *a* be the integral of J_rel_ over 2 APs for I_CaL_ scaling factor of 1. Then, relative J_rel_ integral is *y*/*b*. Integral over 2 APs is used to accommodate for alternans formation. In all cases, the model was paced until steady state for 2500 beats at bcl 260 ms.

A second change to the release properties performed in this work was changing the total amount of calcium released (SR release integral). In order to control this, we used a proxy in the form of scaling I_CaL_ with a multiplier of its conductance, as there is a near–linear relationship between the scaling factor of I_CaL_ and the respective increase in SR release integral in both considered models (**Figures 3A,B**). Scaling I_CaL_ instead of release properties to control total SR release is appealing, as it is possible to maintain the original formulation of SR release. Consequently, any results achieved may be linked to amount of calcium released, as opposed to other changes to the RyR model which could confound the analysis.

## Results

### Mechanisms of Alternans Driven by SR Cycling Refractoriness

Cardiac alternans is generally thought to arise predominantly from the oscillation of calcium subsystem of cardiomyocytes (Weiss et al., 2006). Livshitz and Rudy (2007) have also shown that this is the case in the Rudy-family of models. However, it remains to be elucidated how exactly the models enter the calcium oscillations in the first place.

**FIGURE 4:**
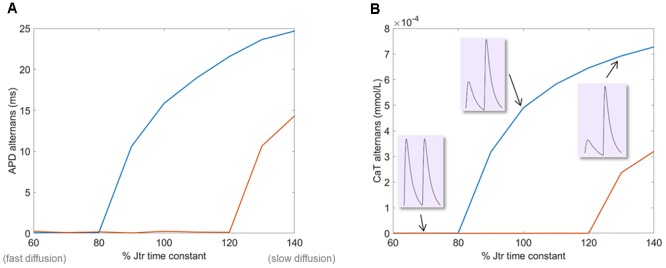
The effect of varying NSR→JSR diffusion time constant on alternans amplitude in the HeRd model. **(A)** The amplitude of APD alternans at two pacing rates: 260 ms (where the baseline model with 100% value of time constant does manifest alternans) and 300 (where the baseline model does not manifest alternans). **(B)** The amplitude of calcium transient (CaT) alternans. In insets are shown examples of calcium transients at the end of the simulation of 260 ms bcl pacing, showing no alternans at 70% time constant, alternans at 100%, and exacerbated alternans at 130%.

We hypothesized that the rate of diffusion from NSR to JSR could be a key factor in alternans formation. Slow diffusion from NSR to JSR could limit timely refilling of JSR and facilitate alternans, as first proposed by Kihara and Morgan (1991). To test this hypothesis, we varied J_tr_, the time constant of the NSR→JSR diffusion in simulated cells, observing the impact on APD and calcium transient alternans. Consistent with the hypothesis, a reduction in the diffusion time constant (corresponding to accelerated NSR→JSR diffusion) attenuated alternans in APD and calcium transient (**Figures 4A,B**). Conversely, increased diffusion time constant promoted alternans formation: Even at 300 ms bcl pacing, when a cell with normal diffusion rate did not manifest alternans, sufficiently diminished diffusion rate (high time constant) allowed alternans to occur.

**FIGURE 5:**
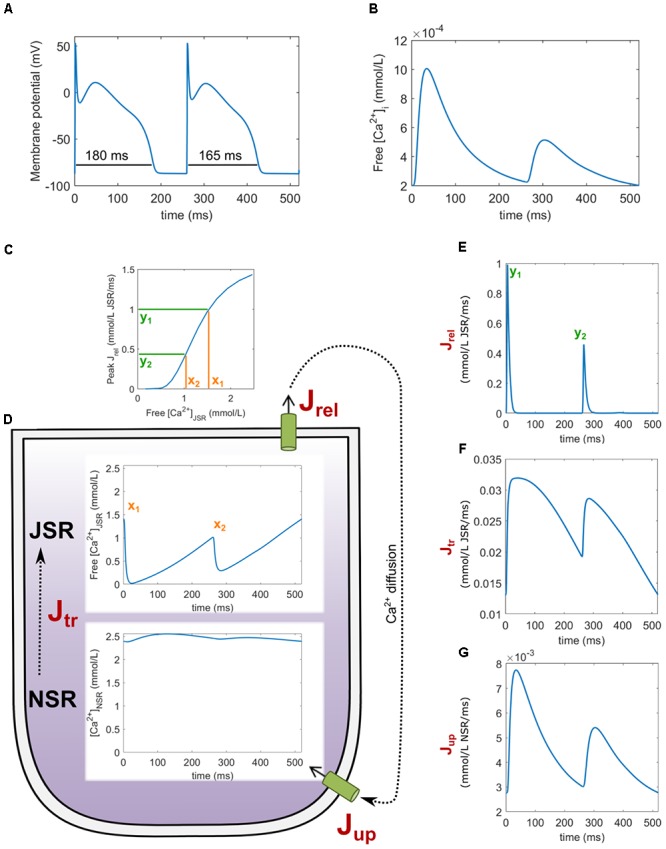
An overview of key variables during alternans in the HeRd model. Two action potentials after reaching the stable state are shown, starting with a prolonged action potential **(A)** with underlying large calcium transient **(B). (C)** The relationship between free JSR calcium and peak J_rel_. **(D)** Free JSR calcium and NSR calcium. Fluxes associated with SR shown are J_rel_ **(E)**, J_tr_ **(F)**, and J_up_ **(G)**. The free Ca^2+^_[JSR]_ levels x_1_, x_2_ and peak release values y_1_, y_2_ are linked with load–release relationship.

We now reconstruct how alternans is sustained, focusing on two consecutive action potentials during stable-state APD and calcium alternans (**Figures 5A,B**). At the start of the first action potential, the JSR calcium concentration is high (x_1_ in **Figure 5D**), leading to a large release J_rel_ (y_1_ in **Figure 5E**). This is followed by calcium diffusion from the junctional subspace to SERCA pumps, allowing for reuptake into NSR (**Figure 5G**). Due to the large depletion of JSR after the first release and a limited rate of diffusion from NSR to JSR (J_tr_, **Figure 5F**), JSR is refilled to a lower value than at the beginning (x_2_ in **Figure 5D**). Crucially, given that the JSR load–release relationship is steep (**Figure 5C**), the following release is small (y_2_ in **Figure 5E**). That is, while the JSR calcium concentration at the start of the first action potential (x_1_) is not drastically higher than at the start of the second one (x_2_), the first peak release y_1_ is approximately two times higher than the second one y_2_. Total amount of calcium released in the first release is 80% higher than in the second one, causing a pronounced difference in JSR depletion. At the same time, the amount of calcium refilled to the JSR is higher only by 23% during the first action potential compared to the second one. The NSR-JSR flux which may be integrated to obtain the amount of calcium refilled is shown in **Figure 5F**. This insensitivity follows from the fact that the NSR→JSR gradient is relatively high at any point during the simulation (**Figure 5D**) and it is not as strongly affected by JSR depletion. Therefore, the JSR refilling is fast enough to restore JSR calcium concentration back to x_1_ within the second AP, perpetuating alternans.

### Dynamics of JSR Release and Alternans: Beyond Amount Released

**FIGURE 6:**
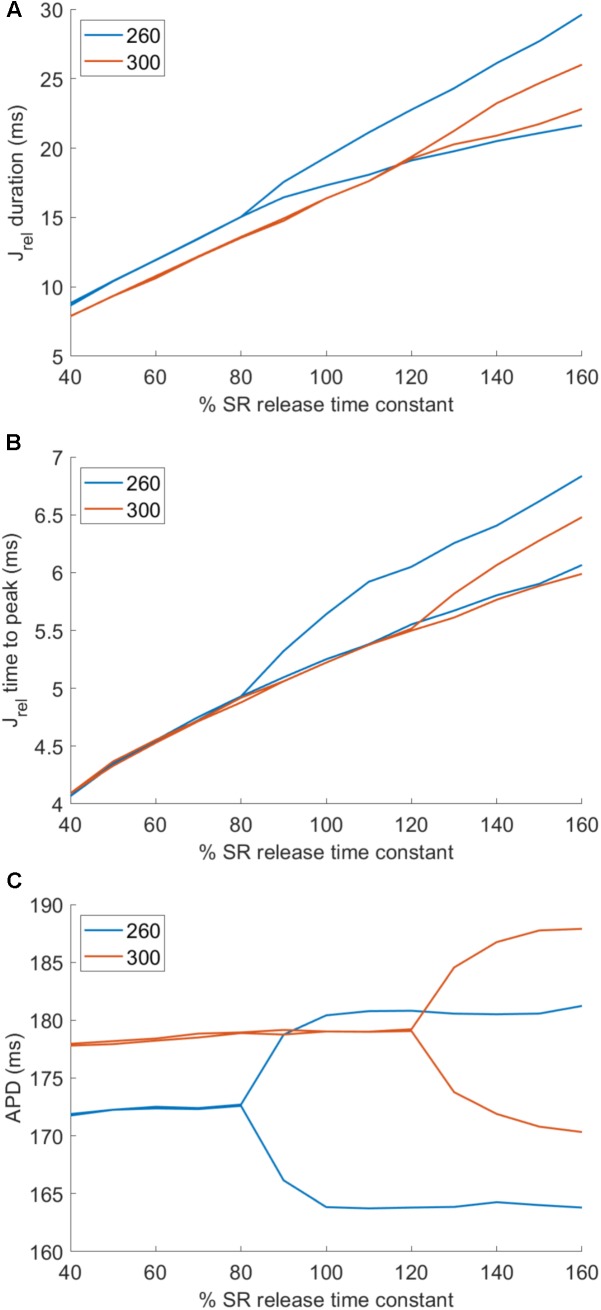
Effect of altering SR release dynamics on alternans at 260 and 300 ms bcl in the HeRd model. Changes in time to peak release **(A)** and duration of release at 90% release recovery **(B)** with scaling of SR release time constant at two pacing rates. The bifurcations correspond to alternans of the respective feature, concurrent with APD alternans **(C)**.

In order to modulate the dynamics of SR release, we modulated the time constant of SR release. Lowering the time constant reduces the time to peak release (**Figure 6A**) and the release duration (**Figure 6B**), while increasing the time constant conversely increases both features of SR release. Crucially, setting the time constant of SR release lower than 80% of the control value abolished alternans in a cell paced at 260 ms bcl (**Figure 6C**). Conversely, sufficiently increased time constant of SR release allowed alternans formation in a cell paced at 300 ms bcl, which does not manifest alternans with baseline value of time constant.

To further separate the influence of shortened time to peak and release duration, we used clamping of SR release to a triangular shape of given parameters (section “Materials and Methods”). We assess the vulnerability of cells with a clamped release based on their ability to refill the JSR to the starting value, following a SR release, as this was shown to be a critical determinant of alternans in the previous section. This indirect approach to quantify alternans vulnerability is based on the fact that clamping the SR, necessary for full control over the release, may prevent alternans formation. For every combination of parameters specifying the shape of the triangular release, we search for the highest total JSR release (stretching the triangle vertically), such that JSR is refilled to its initial value within the action potential (details in section “Materials and Methods”). This amount is termed “alternans threshold”: cells with a high alternans threshold are more resistant to alternans formation than ones with a low alternans threshold, as they can support a larger release without failing to refill the JSR.

**FIGURE 7:**
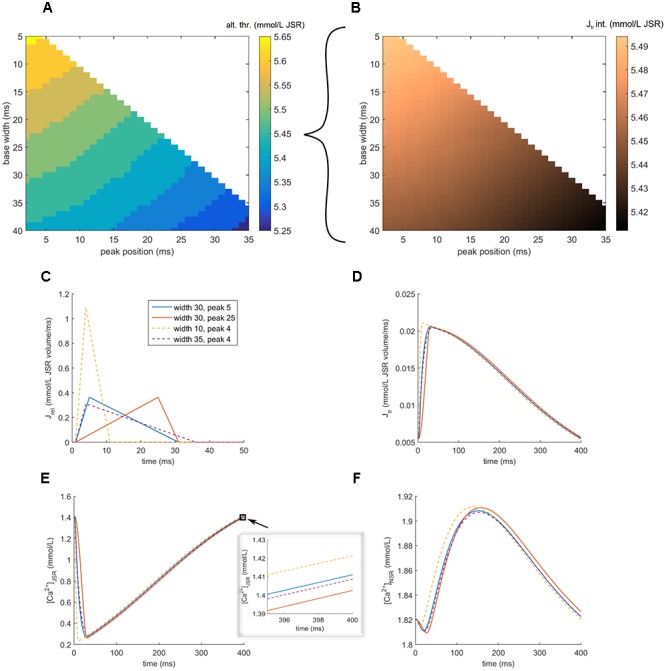
Exploring shapes of SR release and alternans vulnerability in the HeRd model. **(A)** Shown here is the alternans threshold coded by color for various parameters of release triangle. Base width = release duration. Peak time = absolute time after stimulus administration corresponding to time to peak release. Alternans threshold = the level of total calcium released above which a cell with the given release duration and time to peak release fails to refill the JSR to the pre-release state within 400 ms (corresponding to one action potential at the prepacing bcl). **(B)** The integral of J_tr_ (JSR refilling) for a fixed total released calcium of 5.45. **(C)** Shapes of four triangular releases with total released calcium 5.45. The dashed lines vary in release duration, while the solid lines vary in peak position, but have the same duration. **(D)** JSR refilling rate. **(E)** JSR free calcium concentration and an inset of last 5 ms, showing the final calcium concentrations. **(F)** NSR calcium concentration.

We mapped the alternans threshold over a range of pairs of parameters of the SR release (base width, peak time). It is apparent that both shortened release, as well as the earlier peak release improve JSR refilling and are therefore expected to attenuate alternans, confirming the importance of both aspects of release dynamics (**Figure 7A**). Conversely, prolonged release and longer time to peak facilitate alternans formation. Moreover, for a fixed total calcium release, the amount of refilled calcium (J_tr_ integral) increases with both reduced time to peak release and release duration (**Figure 7B**), in line with increased resistance to alternans. For both parameters, the alternans threshold and amount of refilled calcium are affected even when the other parameter is fixed, suggesting their independence.

In the case of earlier time to peak (**Figure 7C**, solid lines; blue vs. red), the key factor is that JSR is depleted sooner (**Figure 7E**, solid lines), providing more time for SR reuptake and JSR refilling, resulting in higher final JSR concentration in the case of earlier-peak release (**Figure 7E**, inset, solid lines). In the case of short-duration release (**Figure 7C**, dashed lines, orange vs. purple), the earlier JSR depletion plays a similar role (**Figure 7E**, dashed lines). Furthermore, such a fast release of high amplitude increases the extra-SR calcium concentration rapidly, enhancing the SERCA reuptake rate and increasing the NSR loading (**Figure 7F**, dashed lines). This increases the NSR→JSR gradient and therefore also the JSR refilling rate J_tr_. Consequently, the final concentration of JSR is higher in the case of shorter release (**Figure 7E**, inset, dashed line). We note that in the case where the calcium fails to refill to the original value in **Figure 7E**, the difference of the starting and final state is small – this corresponds to a very early phase of alternans development, where APD alternans would be also minimal. In a cell developing alternans, the amplitude of JSR release oscillation and thus also diastolic SR loading oscillation self-amplifies to greatly increase over subsequent beats; however, this cannot be shown using a model with clamped SR release. Using an unclamped cell without controlled release shows that during stable-state alternans, the difference between JSR calcium concentration at the start and the end of an action potential is much higher (**Figure 5D**, x_1_ vs. x_2_).

### Increased and Decreased Magnitude of SR Release Attenuates Alternans

**FIGURE 8:**
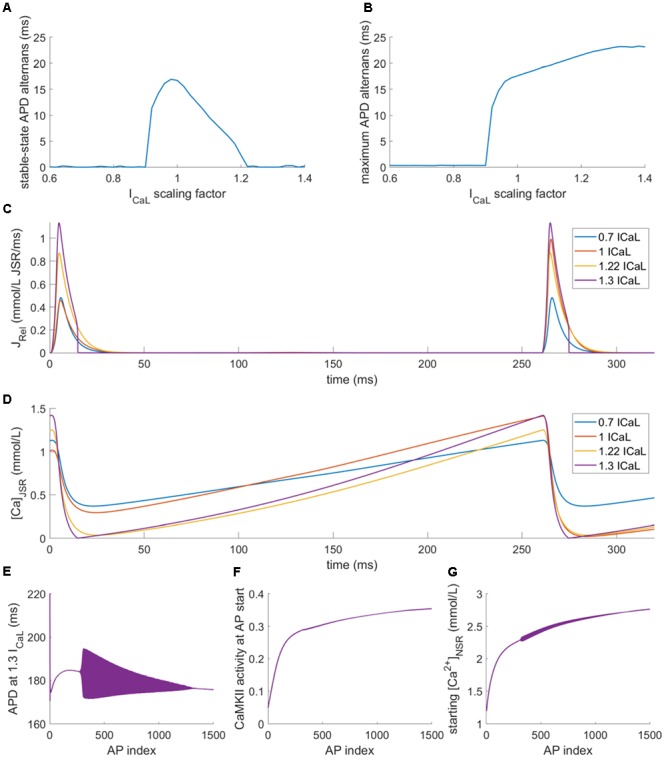
Alternans at 260 ms bcl with varied I_CaL_ conductance in the HeRd model. **(A)** APD alternans amplitude (difference in APD between last two APs) at the end of stable-state pre-pacing. **(B)** Maximum APD alternans (difference in two consecutive APs) at any stage between pre-pacing APs 30–2500 (first 30 APs were omitted as the model may manifest large APD changes at the start of the simulations, which are not due to alternans). **(C)** Examples of J_rel_ for four I_CaL_ scaling factors after stable-state pre-pacing, showing alternans for I_CaL_ scaling factor of 0.7 and 1 but not for 1.22 or 1.3; **(D)** JSR calcium levels aligned with the plot **(C),** showing full depletion in both beats at calcium scaling factors of 1.22 and 1.3. **(E)** Development of APD during pre-pacing for I_CaL_ scaling factor of 1.3, the filled area represents alternans of APD; first 1500 beats of pre-pacing shown. **(F)** Increasing CaMKII activity during pre-pacing for I_CaL_ scaling factor of 1.3. **(G)** Increasing NSR loading during pre-pacing of the model with I_CaL_ scaling factor of 1.3, measured as NSR calcium at the start of an action potential.

In the previous section, we characterized how altered release dynamics modulate alternans, given a fixed calcium release magnitude. In this section, we instead maintain given dynamics and study the effect of varying amount of released calcium. We scaled the conductance of the I_CaL_ current in order to manipulate the SR release magnitude within an action potential (see section “Materials and Methods” for details and validation of near–linear relationship between I_CaL_ conductance and SR release magnitude). The baseline model with control SR release magnitude manifests APD alternans of 17 ms amplitude. In cells with sufficiently decreased SR release (I_CaL_ scaling factor <0.9), the total SR release was inhibited and no alternans was present (**Figure 8A**). This is to be expected: given that alternans in the model arises from an overtly large JSR depletion which cannot be refilled, lowering the SR release overall prevents this event from occurring.

Surprisingly, also sufficiently increased SR release (I_CaL_ scaling factor >1.2) abolished alternans in the stable state after 2500 beats (**Figure 8A**). This is a less expected result: since alternans is driven by SR release oscillations, potentiation of the release might be expected to increase the alternans magnitude. We note that scaling I_CaL_ produced only small effects in J_rel_ dynamics, such as up to 10% change in J_rel_ time to peak. According to results in **Figure 7**, this would have negligible effects on alternans vulnerability, particularly for J_rel_ time to peak of 5 ms as in the ORd/HRd models. Therefore, the effects of I_CaL_ scaling on alternans vulnerability in **Figure 8** are mainly due to alterations in J_rel_ magnitude as quantified in **Figure 3**.

We noticed that alternans did occur in models with increased SR release, but only transiently, i.e., before reaching the stable state (**Figures 8B,E**). Interestingly, after reaching the stable state, JSR was fully depleted in every beat. For intermediate increase in I_CaL_ conductance (scaling factor 1.22, yellow trace in **Figures 8C,D**), the decay of J_rel_ is smooth, similar to the case of cells with low or normal SR release **(Figures 8C,D**). However, for large increase in I_CaL_ conductance (scaling factor 1.3, purple trace in **Figures 8C,D**), the SR release drops to zero abruptly. We are not aware of an experimental study which would report similarly abrupt termination of the SR release and this could be an artifact of the model. It follows from a highly accelerated convergence of the gate variable determining J_rel_ to its steady-state value of zero release when J_rel_ is high, but the JSR loading is low. However, as described below, the aspect crucial for alternans abolishment is the fact of depletion, not of the abrupt termination of release.

The full depletion of JSR leads to disappearance of alternans predominantly by a combination of two factors. The first factor is that when all releasable calcium in the JSR is released, the otherwise steep load–release relationship is flattened: when a JSR is depleted during a release, increasing the initial JSR loading further does not translate into considerably increased release given that there is no additional calcium to be released. Second, the refilling of JSR is rapid enough to reach the starting JSR concentration, allowing again for a full depletion in the next AP. During the pacing shown in **Figure 8E**, the CaMKII activity gradually rose (**Figure 8F**), increasing the reuptake rate of SERCA pumps, translating into high NSR loading (**Figure 8G**). In addition, the total reuptake is generally increased in models with high I_CaL_ scaling, given that more calcium enters the cell. The increased NSR loading consequently increases the JSR refilling rate and allows maintaining the depletion of the SR in the stable state.

It can be shown that the mechanism of alternans abolishment via large SR release underlies the difference in alternans vulnerability between different variants (epicardial, endocardial, and midmyocardial) of the highly popular human cardiomyocyte ORd model (O’Hara et al., 2011) used previously to study alternans (Zhou et al., 2016). In this model, only the endocardial variant is able to reproduce alternans in stable state.

**FIGURE 9:**
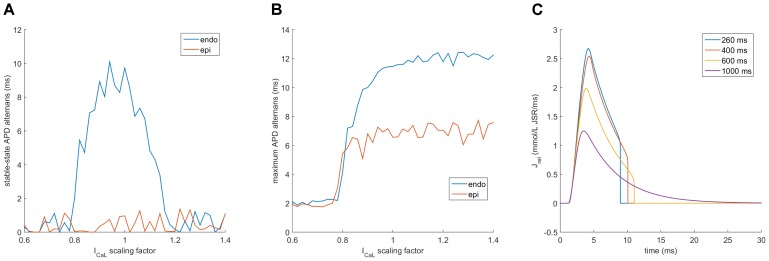
Alternans with varied I_CaL_ conductance in the ORd model. **(A,B)** are an analogy of **Figures 8A,B** obtained using the ORd model paced at 260 ms bcl, showing the data for both endocardial (endo) and epicardial (epi) variant, **(C)** shows traces of J_rel_ in the epicardial variant of ORd after 2500 pre-pacing beats at four different base cycle lengths. The fluctuation in APD alternans amplitude in panels **(A,B)** are due to a higher APD variability of the ORd model at rapid pacing, compared to HeRd.

We repeated the protocols testing the impact of I_CaL_ modulation on alternans formation in all three variants of the ORd model. Midmyocardial cells generally manifested behavior akin to 2:1 block at fast pacing and did not develop stable behavior, which is why they were excluded from further analysis. While the endocardial variant of ORd developed stable-state alternans for similar values of I_CaL_ modifier as HeRd (extended toward lower values), the epicardial variant was immune to alternans in the stable state altogether (**Figure 9A**). However, observing the maximum alternans amplitude during pre-pacing (**Figure 9B**) revealed that alternans did develop in the epicardial variant of ORd, but only transiently (the baseline alternans amplitude of 2 ms for low I_CaL_ modifier reflects natural APD variability in the ORd model), in a similar fashion to HeRd or endocardial ORd model with highly increased I_CaL_. Plotting SR release for different bcls in the epicardial ORd variant, it can be clearly seen that up to 600 ms bcl (heart rate of 100 bpm, i.e., relatively normal), SR release becomes zero abruptly, marking functional JSR depletion (**Figure 9C**). Among the differences between the endocardial and epicardial variants of ORd is the increased I_CaL_ conductance and increased SERCA reuptake in the epicardial model, from which the susceptibility to JSR depletion follows naturally.

**FIGURE 10:**
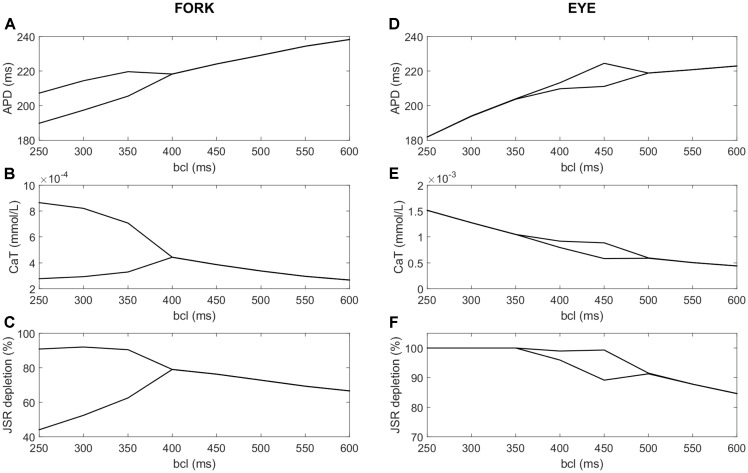
A comparison of fork and eye-type alternans in the ORd model. The left column shows fork alternans in APD **(A)** and calcium transient amplitude **(B)**, with underlying fractional JSR depletion per action potential **(C)**, computed as: 
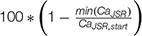
, where min(Ca_JSR_) is the minimum JSR contents per action potential and *Ca_JSR, start_* is the starting JSR calcium concentration in the same action potential. In panels **(D–F)** are respectively shown APD, CaT, and depletion in eye-type alternans. All plots show the values of the two last action potentials for every bcl and variable of interest; bifurcation indicates that the last two action potentials differ in the variable of interest, manifesting alternans.

Understanding the importance of full JSR depletion may be applied to gain insight the disappearance of alternans at rapid pacing in so called “eye-type” alternans, investigated previously by Zhou et al. (2016) using the ORd model. The study used a population of models approach to study the differences between cells manifesting “fork-type” alternans, where the APD bifurcation opens at a certain pacing frequency and is present at all faster frequencies (**Figures 10A–C**), and “eye-type alternans,” where the bifurcation opens at a given pacing frequency, but then closes again as the pacing frequency increases (**Figures 10D–F**). A population of ORd model cells based on the epicardial variant with varying channel conductances was simulated. Out of the 2326 simulated cells, 87 manifested alternans: 14 of the eye type and 73 of the fork type. The proposed key difference between cells manifesting these two subtypes of alternans was I_CaL_ conductance. All details of the methodology are given in Zhou et al. (2016).

We repeated the simulation of the population as described in the article, measuring JSR depletion in the last two action potentials, as well as the APD90 and calcium transient. We hypothesized that the functional depletion of JSR may contribute to the eye closing at fast pacing frequencies. Consistent with the hypothesis, we observed that in all the 14 cases of eye-type alternans, the eye closure occurred at the same bcl as the functional JSR depletion in both recorded action potentials. At the same time, in no case of fork alternans did full functional depletion occur in both action potentials. **Figures 10A–C** shows a representative APD, calcium transient, and relative JSR depletion for the fork-type alternans. Corresponding visualization of the given features in eye-type alternans is given in **Figures 10D–F**. Reducing the bcl beyond 500 ms increases the alternans in amplitude both in APD (**Figure 10D**) and calcium transient (**Figure 10E**). However, at 450 ms bcl, one of the two last action potentials reaches full functional JSR depletion (**Figure 10F**), preventing further divergence of SR release in consecutive beats. With further decreasing bcl, JSR calcium release keeps increasing in the other beat, “catching up” the larger of the two releases. Subsequently, when JSR is fully depleted in both action potentials at bcl 350 ms, the alternans eye closes (**Figures 10D,E**).

## Discussion

The main findings of this work are: (1) Accelerated dynamics of SR release attenuate alternans via acceleration of the junctional SR refilling, while slow SR release promotes alternans. (2) A sufficient increase in the magnitude of SR release may attenuate alternans, potentially explaining the formation of “eye-type” alternans. (3) Alternans in the studied models is mainly driven by refractoriness of calcium cycling and junctional SR refilling, supporting SR calcium cycling refractoriness (SRCCR) as a mechanism for alteranans.

### Calcium Driven by SR Cycling Refractoriness

Numerous previous studies have investigated the subcellular determinants of calcium transient and repolarization alternans (Qu et al., 2000; Fox et al., 2002; Picht et al., 2006; Weiss et al., 2006; Livshitz and Rudy, 2007). In general, these studies have identified a critical role for SR calcium-cycling properties in calcium transient alternans development, focusing either on refractoriness of release via slow reactivation of RyR (Picht et al., 2006), or slow calcium reuptake resulting in a release-reuptake mismatch (Weiss et al., 2006). Our work highlights the potential importance of a third explanation, “SR calcium cycling refractoriness” (SRCCR).

The explanation of alternans presented in this article relies primarily on steepness of load–release relationship combined with limited rate of diffusion between NSR and JSR preventing timely refilling of releasable calcium. The importance of NSR→JSR diffusion in alternans was first suggested by Kihara and Morgan (1991) and it underlies the alternans formation in the Mahajan model (Mahajan et al., 2008). This process may be enabled by low mobility of calcium diffusing from reuptake to release sites (Swietach et al., 2008). The SRCCR-driven alternans bears similarities with the two major explanations (release-reuptake mismatch and RyR refractoriness), but is distinct at the same time. Similarly to the hypothesis of release-reuptake mismatch, SR calcium oscillations caused by SRCCR play an important role in alternans generation. On the other hand, the release-reuptake hypothesis does not include any form of SR release refractoriness (whether driven by JSR refilling or intrinsic RyR refractoriness), shown to be crucial in alternans generation (Shkryl et al., 2012). Both hypotheses therefore offer a slightly different point of view on the experimental observation that the upregulation of SERCA pumps and thus improved reuptake attenuates alternans (Cutler et al., 2012; Stary et al., 2016).

In the release-reuptake mismatch hypothesis of alternans, upregulation of SERCA pumps directly corrects the mismatch between release and reuptake, however, the explanation is based on an implicit assumption that the reuptaken calcium is immediately releasable. In models representing intra-SR diffusion of calcium, it can be seen that even if all released calcium is reuptaken, it might not diffuse from the reuptake to the release site in time, failing to prevent alternans. Instead, in the more advanced model including intra-SR diffusion, increased expression of SERCA pumps may be seen as a way of increasing NSR loading. This subsequently translates into larger gradient between NSR and JSR and thus faster NSR→JSR transport and consequently accelerated JSR refilling (reducing release refractoriness). This then attenuates alternans, given alternans formation in SRCCR hypothesis is directly linked to the JSR refilling, rather than SR reuptake.

The SRCCR-driven alternans presented here is similar to the hypothesis on RyR refractoriness driving alternans (Picht et al., 2006) in that both models rely on a form of SR refractoriness. The mechanism of refractoriness is however different. Picht et al. observed alternans without oscillations of diastolic SR calcium in 20% of cells studied. This is hard to explain using the SRCCR alternans mechanism, and thus suggests the existence of an intrinsic RyR refractoriness instead. Consistently with this, Ramay et al. (2011) observed that the frequency of calcium sparks (discrete SR release events) may be increased (via caffeine) or decreased (via tetracaine), supporting the existence of intrinsic RyR refractoriness. At the same time, however, RyR reopening soon after the first opening was associated with considerably diminished spark amplitude, and RyR sensitization via caffeine did not affect its recovery. Even 300 ms after the previous calcium spark, the amplitude of the following calcium spark at a given location was not fully recovered, showing that the time scale of recovery is consistent with a role in alternans during rapid pacing. Similar results were observed in a study by Sobie et al. (2005). This suggests that local JSR refilling might play an important role in refractoriness of overall SR release. In addition, the study by Szentesi et al. (2004) showed that increased SR reuptake accelerates the recovery of SR release from refractoriness. While this is highly consistent with accelerated JSR refilling downstream of altered SR reuptake, it is not immediately obvious how increased SR reuptake would modulate the intrinsic RyR refractoriness. It therefore appears that refractoriness of SR release may be driven both by intrinsic RyR refractoriness and by JSR refilling refractoriness. Taken together, the literature suggests that the two types of refractoriness are not mutually exclusive and that both are physiologically relevant.

### Release Dynamics Modulate Alternans Occurrence

Using manipulation of SR release time constant and using a synthetic clamping of SR release, we have shown that accelerating release dynamics improves JSR refilling and acts against alternans formation. These results are linked to our previous work (Tomek et al., 2017), where we have shown that alternans is attenuated when RyR are phosphorylated by β-adrenergic stimulation. The key feature of RyR phosphorylation in this model was altered release dynamics: a shorter-duration release and a shorter time to peak release.

We show that reducing the release time constant (shortening both time to peak release and release duration) attenuates alternans, while increasing the time constant promotes it. In addition, we used a well-controlled SR release clamping approach to show that both changes to dynamics contribute to reduction in alternans independently, explaining why they improve JSR refilling. Conversely, our results show that the combination of JSR release prolongation, time-to-peak prolongation, and amplitude reduction promotes alternans. This could provide a novel explanation of increased alternans vulnerability in cardiac diseases, such as heart failure (Kodama et al., 2004), hypertrophic cardiomyopathy (Cannon et al., 1986), or myocardial infarction (Gardner et al., 2015).

In heart failure, reduced release rate was observed directly by Yamamoto et al. (1999). Moreover, longer time-to-peak, slower decay, and reduced amplitude of calcium sparks were observed (Lindner et al., 2002). Calcium sparks are microscopic releases of calcium from JSR, the sum of which constitutes total SR release at a given time (Collier et al., 2000). Such dynamics of calcium sparks observed in heart failure therefore correspond precisely to the phenotype of longer release, longer time-to-peak, and reduced amplitude, which is expected to be vulnerable to alternans according to our model. Recently, Zhong et al. (2018) similarly observed that reduced expression of ryanodine receptors prolonged the SR release, and that this promoted alternans formation in the hearts.

A further link between alternans vulnerability and release dynamics may be established via the activity of CaMKII, which increases the duration of calcium sparks, prolonging calcium release (Maier et al., 2003; Guo et al., 2006). At the same time, activity of CaMKII is increased in hypertrophy and heart failure (Anderson et al., 2011), as well as within the cardiac post-infarction border zone (Hund et al., 2008), which is a region vulnerable to alternans (Gardner et al., 2015). Therefore, we can conclude that the prolongation of SR release might be contributing to alternans vulnerability in a wide range of cardiac diseases.

We believe that the importance of modulation of SR release dynamics is not limited to the presented mechanism of alternans formation. In the case of alternans driven by the refractoriness of the ryanodine receptors (Picht et al., 2006), early opening of RyR, corresponding to accelerated SR release, provides all ryanodine receptors with more time to recover from refractoriness before the subsequent action potential. Conversely, slow SR release requires late opening of ryanodine receptors, which then have less time to recover, increasing the likelihood of them being refractory at the start of the following action potential, promoting alternans.

### Calcium Amount Released and Alternans

In this study, we have shown that sufficiently strong inhibition or potentiation of calcium release can protect the calcium subsystem from alternans. Under inhibited calcium release, the amount of calcium released via RyR is reduced, so that enough JSR calcium can be refilled in time during action potential. In the case of potentiated calcium release, alternans is abolished due to two factors: functional depletion of JSR in every action potential, flattening the steep load–release relationship, and allowing for rapid JSR refilling through the increased NSR loading.

This phenomenon of full JSR depletion is relevant in understanding the principle of formation of eye-type alternans (APD bifurcation which opens with increasing pacing frequency, then disappearing with further increase in frequency), recently investigated in human data and simulations by Zhou et al. (2016). We show that the disappearance of alternans at fastest pacing frequencies in the eye-type alternans may be caused by functional depletion of JSR in every beat to eliminate alternans at fast pacing. The fact that the eye-type alternans cells in the original study tend to have a higher L-type calcium current conductance compared to fork-type is consistent with our results. First of all, the stronger L-type calcium current translates into higher RyR release, which facilitating functional depletion of JSR at rapid pacing and eye closing. Secondly, the stronger calcium influx mediated via increased L-type calcium current contributes to higher diastolic calcium level, increased SR reuptake, and faster calcium refilling to JSR, allowing maintenance of full JSR release in every beat.

More experimental evidence is needed to clarify the JSR calcium load–release relationship during alternans and whether full functional JSR depletion may occur. Current evidence suggests that functional JSR depletion in myocytes is only 60–80% at most (Bassani et al., 1995; Shannon et al., 2000), even during alternans (Shkryl et al., 2012). However, it is possible that the cells studied in these works represent the fork-type alternans behavior rather than the eye-type. Moreover, it was observed that caffeine-driven release depletes JSR more than a normal release driven by elevated calcium concentration (MacQuaide et al., 2009); it is therefore possible that using caffeine to estimate JSR contents overestimates calcium releasable in a physiological way. Consequently, an 80% depletion of JSR as assessed by caffeine may in fact represent full functional depletion. It is therefore not clear whether the full depletion of JSR in the studied computer models is physiological or if this suggests that the models require further development in this regard. Investigating the slope of JSR load–release relationship in myocytes with a wide range of external calcium stimuli (achievable acutely, e.g., via caged calcium) represents an interesting experiment which could elucidate whether full functional depletion may occur at rapid pacing in myocytes. A potentially important factor to be investigated may be the role of calcium buffering by calsequestrin in the load–release relationship. A recent study suggests that JSR depletion might result in change of calsequestrin conformation and a subsequent closure of the ryanodine receptors (Manno et al., 2017). This provides a mechanism of SR release termination, with possibly similar effect to SR release termination due to JSR depletion.

An indication that the load-release curve may become flatter at high SR contents is provided by the work of Posterino and Lamb (2003) in rat fast-twitch skeletal muscle. The authors show that after the initial steep phase of load–release relationship, increasing SR calcium loading does not translate into an increased SR release (Posterino and Lamb, 2003; Figures 10B,C), flattening the load–release relationship. While it is not explicitly shown that the flattening occurs via functional depletion of JSR, this nevertheless could explain the disappearance of alternans at fast pacing rates in a similar fashion to our depletion-based explanation: at intermediate pacing frequencies where the load–release relationship is steep, alternans may occur. However, with increasing pacing frequency, the load-release flattening may dampen the JSR release oscillation, abolishing alternans and closing the alternans eye. The degree of SR loading at rapid pacing would then be the key feature determining whether a cell manifests alternans of the fork or the eye type.

The eye-type alternans was previously observed also in a mathematical study by Qu et al. (2007). In this work, the closure of the eye at fast pacing rates was linked to rate-dependent attenuation of the function corresponding to the load–release relationship at the very fast pacing rates, assuming rate-independent reuptake. The functional JSR depletion at the fastest pacing rates described in our article may be linked to this mechanism. In our simulations, the reuptake is not rate-independent, but increases with pacing rate (mainly via CaMKII signaling). While the load–release relationship is not strictly reduced at the highest pacing rates, its growth is considerably attenuated by the JSR depletion. This would presumably allow the rate-activated SR reuptake to “overtake” the plateauing SR release, achieving a similar effect to a rate-independent reuptake and rate-inhibited release.

## Author Contributions

JT conceived and designed most of the study, performed the simulations and analyses, and wrote most of the manuscript. MT contributed to the design of simulations, figure design, and manuscript writing. XZ aided JT with reuse of codes from her previous study and contributed to the manuscript writing. GB and BR supervised the project and strongly contributed to the manuscript writing and structuring.

## Conflict of Interest Statement

The authors declare that the research was conducted in the absence of any commercial or financial relationships that could be construed as a potential conflict of interest.

## Footnotes

1 http://rudylab.wustl.edu/research/cell/code/AllCodes.html

## References

[B1] Anderson M. E. Brown J. H. Bers D. M. (2011). CaMKII in myocardial hypertrophy and heart failure. *J. Mol. Cell. Cardiol.* 51 468–473. 10.1016/j.yjmcc.2011.01.012 21276796 PMC3158288

[B2] Bassani J. W. Yuan W. Bers D. M. (1995). Fractional SR Ca release is regulated by trigger Ca and SR Ca content in cardiac myocytes. *Am. J. Physiol.* 268(5 Pt 1), C1313–C1319. 10.1152/ajpcell.1995.268.5.C1313 7762626

[B3] Bayer J. D. Lalani G. G. Vigmond E. J. Narayan S. M. Trayanova N. A. (2016). Mechanisms linking electrical alternans and clinical ventricular arrhythmia in human heart failure. *Heart Rhythm* 13 1922–1931. 10.1016/j.hrthm.2016.05.017 27215536 PMC4996715

[B4] Cannon R. O. Schenke W. H. Bonow R. O. Leon M. B. Rosing D. R. (1986). Left ventricular pulsus alternans in patients with hypertrophic cardiomyopathy and severe obstruction to left ventricular outflow. *Circulation* 73 276–285. 10.1161/01.CIR.73.2.276 3943162

[B5] Collier M. L. Ji G. Wang Y.-X. Kotlikoff M. I. (2000). Calcium-induced calcium release in smooth muscle - loose coupling between the action potential and calcium release. *J. Gen. Physiol.* 115 653–662. 10.1085/jgp.115.5.653 10779321 PMC2217224

[B6] Cutler M. J. Wan X. Plummer B. N. Liu H. Deschenes I. Laurita K. R. (2012). Targeted sarcoplasmic reticulum Ca^2+^ ATPase 2a gene delivery to restore electrical stability in the failing heart. *Circulation* 126 2095–2104. 10.1161/CIRCULATIONAHA.111.071480 23019291 PMC3538142

[B7] Decker K. F. Heijman J. Silva J. R. Hund T. J. Rudy Y. (2009). Properties and ionic mechanisms of action potential adaptation, restitution, and accommodation in canine epicardium. *AJP Heart Circ. Physiol.* 296 H1017–H1026. 10.1152/ajpheart.01216.2008 19168720 PMC2670702

[B8] Díaz M. E. O’Neill S. C. Eisner D. A. (2004). Sarcoplasmic reticulum calcium content fluctuation is the key to cardiac alternans. *Circ. Res.* 94 650–656. 10.1161/01.RES.0000119923.64774.72 14752033

[B9] Edwards J. N. Blatter L. A. (2014). Cardiac alternans and intracellular calcium cycling. *Clin. Exp. Pharmacol. Physiol.* 41 524–532. 10.1111/1440-1681.12231 25040398 PMC4122248

[B10] Fox J. J. McHarg J. L. Gilmour R. F. (2002). Ionic mechanism of electrical alternans. *Am. J. Physiol. Heart Circ. Physiol.* 282 H516–H530. 10.1152/ajpheart.00612.2001 11788399

[B11] Gardner R. T. Wang L. Lang B. T. Cregg J. M. Dunbar C. L. Woodward W. R. (2015). Targeting protein tyrosine phosphatase sigma after myocardial infarction restores cardiac sympathetic innervation and prevents arrhythmias. *Nat. Commun.* 6:6235. 10.1038/ncomms7235 25639594 PMC4315356

[B12] Guo T. Zhang T. Mestril R. Bers D. M. (2006). Ca^2+^/calmodulin-dependent protein kinase II phosphorylation of ryanodine receptor does affect calcium sparks in mouse ventricular myocytes. *Circ. Res.* 99 398–406. 10.1161/01.RES.0000236756.06252.13 16840718

[B13] Heijman J. Volders P. G. Westra R. L. Rudy Y. (2011). Local Control of β-adrenergic stimulation: effects on ventricular myocyte electrophysiology and Ca^2+^-transient. *J. Mol. Cell. Cardiol.* 50 863–871. 10.1016/j.yjmcc.2011.02.007 21345340 PMC3075371

[B14] Hund T. J. Decker K. F. Peter E. K. Mohler J. Boyden P. A. Schuessler R. B. (2008). Role of activated CaMKII in abnormal calcium homeostasis and INA remodeling after myocardial infarction: insights from mathematical modeling. *J. Mol. Cell. Cardiol.* 45 420–428. 10.1016/j.yjmcc.2008.06.007 18639555 PMC2587155

[B15] Hund T. J. Rudy Y. (2004). Rate dependence and regulation of action potential and calcium transient in a canine cardiac ventricular cell model. *Circulation* 110 3168–3174. 10.1161/01.CIR.0000147231.69595.D3 15505083 PMC1851913

[B16] Kanaporis G. Blatter L. A. (2015). The mechanisms of calcium cycling and action potential dynamics in cardiac alternans. *Circ. Res.* 116 846–856. 10.1161/CIRCRESAHA.116.305404 25532796 PMC4344847

[B17] Kho C. Lee A. Hajjar R. J. (2012). Altered sarcoplasmic reticulum calcium cycling—targets for heart failure therapy. *Nat. Rev. Cardiol.* 9 717–733. 10.1038/nrcardio.2012.145 23090087 PMC3651893

[B18] Kihara Y. Morgan J. P. (1991). Abnormal Ca_i_^2+^ handling is the primary cause of mechanical alternans: study in ferret ventricular muscles. *Am. J. Physiol.* 261(6 Pt 2), H1746–H1755. 10.1152/ajpheart.1991.261.6.H1746 1750531

[B19] Kodama M. Kato K. Hirono S. Okura Y. Hanawa H. Yoshida T. (2004). Linkage between mechanical and electrical alternans in patients with chronic heart failure. *Cardiovasc. Electrophysiol.* 15 295–299. 10.1046/j.1540-8167.2004.03016.x 15030419

[B20] Lehnart S. E. Terrenoire C. Reiken S. Wehrens X. H. T. Song L.-S. Tillman E. J. (2006). Stabilization of cardiac ryanodine receptor prevents intracellular calcium leak and arrhythmias. *Proc. Natl. Acad. Sci. U.S.A.* 103 7906–7910. 10.1073/pnas.0602133103 16672364 PMC1472543

[B21] Lindner M. Brandt M. C. Sauer H. Hescheler J. Böhle T. Beuckelmann D. J. (2002). Calcium sparks in human ventricular cardiomyocytes from patients with terminal heart failure. *Cell Calcium* 31 175–182. 10.1054/ceca.2002.0272 12027382

[B22] Livshitz L. M. Rudy Y. (2007). Regulation of Ca^2+^ and electrical alternans in cardiac myocytes: role of CaMKII and repolarizing currents. *Am. J. Physiol. Heart Circ. Physiol.* 292 H2854–H2866. 10.1152/ajpheart.01347.2006 17277017 PMC2274911

[B23] MacQuaide N. Dempster J. Smith G. L. (2009). Assessment of sarcoplasmic reticulum Ca^2+^ depletion during spontaneous Ca^2+^ waves in isolated permeabilized rabbit ventricular cardiomyocytes. *Biophys. J.* 96 2744–2754. 10.1016/j.bpj.2008.12.3944 19348757 PMC2711300

[B24] Mahajan A. Shiferaw Y. Sato D. Baher A. Olcese R. Xie L. H. (2008). A rabbit ventricular action potential model replicating cardiac dynamics at rapid heart rates. *Biophys. J.* 94 392–410. 10.1529/biophysj.106.98160 18160660 PMC2157228

[B25] Maier L. S. Zhang T. Chen L. DeSantiago J. Brown J. H. Bers D. M. (2003). Transgenic CaMKIIδc overexpression uniquely alters cardiac myocyte Ca^2+^ handling: reduced SR Ca^2+^ load and activated SR Ca^2+^ release. *Circ. Res.* 92 904–911. 10.1161/01.RES.0000069685.20258.F1 12676813

[B26] Manno C. Figueroa L. C. Gillespie D. Fitts R. Kang C. Franzini-Armstrong C. (2017). Calsequestrin depolymerizes when calcium is depleted in the sarcoplasmic reticulum of working muscle. *Proc. Natl. Acad. Sci. U.S.A.* 114 E638–E647. 10.1073/pnas.1620265114 28069951 PMC5278470

[B27] Narayan S. M. Franz M. R. Clopton P. Pruvot E. J. Krummen D. E. (2011). Repolarization alternans reveals vulnerability to human atrial fibrillation. *Circulation* 123 2922–2930. 10.1161/CIRCULATIONAHA.110.977827 21646498 PMC3135656

[B28] O’Hara T. Virág L. Varró A. Rudy Y. (2011). Simulation of the undiseased human cardiac ventricular action potential: model formulation and experimental validation. *PLoS Comput. Biol.* 7:e1002061. 10.1371/journal.pcbi.1002061 21637795 PMC3102752

[B29] Pastore J. M. Girouard S. D. Laurita K. R. Akar F. G. Rosenbaum D. S. (1999). Mechanism linking T-wave alternans to the genesis of cardiac fibrillation. *Circulation* 99 1385–1394. 10.1161/01.CIR.99.10.1385 10077525

[B30] Picht E. DeSantiago J. Blatter L. A. Bers D. M. (2006). Cardiac Alternans do not rely on diastolic sarcoplasmic reticulum calcium content fluctuations. *Circ. Res.* 99 740–748. 10.1161/01.RES.0000244002.88813.91 16946134

[B31] Posterino G. S. Lamb G. D. (2003). Effect of sarcoplasmic reticulum Ca^2+^ content on action potential-induced Ca^2+^ release in rat skeletal muscle fibres. *J. Physiol.* 551 219–237. 10.1113/jphysiol.2003.040022 12844504 PMC2343158

[B32] Pruvot E. J. Katra R. P. Rosenbaum D. S. Laurita K. R. (2004). Role of calcium cycling versus restitution in the mechanism of repolarization alternans. *Circ. Res.* 94 1083–1090. 10.1161/01.RES.0000125629.72053.95 15016735

[B33] Qu Z. Garfinkel A. Chen P.-S. Weiss J. N. (2000). Mechanisms of discordant alternans and induction of reentry in simulated cardiac tissue. *Circulation* 102 1664–1670. 10.1161/01.CIR.102.14.1664 11015345

[B34] Qu Z. Liu M. B. Nivala M. (2016). A unified theory of calcium alternans in ventricular myocytes. *Sci. Rep.* 6:35625. 10.1038/srep35625 27762397 PMC5071909

[B35] Qu Z. Shiferaw Y. Weiss J. N. (2007). Nonlinear dynamics of cardiac excitation-contraction coupling: an iterated map study. *Phys. Rev. E Stat. Nonlin. Soft Matter Phys.* 75(1 Pt 1), 011927. 10.1103/PhysRevE.75.011927 17358204

[B36] Ramay H. R. Liu O. Z. Sobie E. A. (2011). Recovery of cardiac calcium release is controlled by sarcoplasmic reticulum refilling and ryanodine receptor sensitivity. *Cardiovasc. Res.* 91 598–605. 10.1093/cvr/cvr143 21613275 PMC3156908

[B37] Shannon T. R. Ginsburg K. S. Bers D. M. (2000). Potentiation of fractional sarcoplasmic reticulum calcium release by total and free intra-sarcoplasmic reticulum calcium concentration. *Biophys. J.* 78 334–343. 10.1016/S0006-3495(00)76596-76599 10620297 PMC1300641

[B38] Shkryl V. M. Maxwell J. T. Domeier T. L. Blatter L. A. (2012). Refractoriness of sarcoplasmic reticulum Ca^2+^ release determines Ca^2+^ alternans in atrial myocytes. *AJP Heart Circ. Physiol.* 302 H2310–H2320. 10.1152/ajpheart.00079.2012 22467301 PMC3378301

[B39] Sobie E. A. Song L.-S. Lederer W. J. (2005). Local recovery of Ca^2+^ release in rat ventricular myocytes. *J. Physiol.* 565(Pt 2), 441–447. 10.1113/jphysiol.2005.086496 15817631 PMC1464523

[B40] Stary V. Puppala D. Scherrer-Crosbie M. Dillmann W. H. Armoundas A. A. (2016). SERCA2a upregulation ameliorates cellular alternans induced by metabolic inhibitions. *J. Appl. Physiol.* 120 865–875. 10.1152/japplphysiol.00588.2015 26846549 PMC4835906

[B41] Swietach P. Spitzer K. W. Vaughan-Jones R. D. (2008). Ca^2+^-mobility in the sarcoplasmic reticulum of ventricular myocytes is low. *Biophys. J.* 95 1412–1427. 10.1529/biophysj.108.130385 18390622 PMC2479570

[B42] Szentesi P. Pignier C. Egger M. Kranias E. G. Niggli E. (2004). Sarcoplasmic reticulum Ca^2+^ refilling controls recovery from Ca^2+^-induced Ca^2+^ release refractoriness in heart muscle. *Circ. Res.* 95 807–813. 10.1161/01.RES.0000146029.80463.7d 15388639

[B43] Tomek J. Rodriguez B. Bub G. Heijman J. (2017). β-adrenergic receptor stimulation inhibits proarrhythmic alternans in post-infarction border zone cardiomyocytes: a computational analysis. *Am. J. Physiol. Heart Circ. Physiol.* 313 H338–H353. 10.1152/ajpheart.00094.2017 28550171 PMC5582914

[B44] Weiss J. N. Karma A. Shiferaw Y. Chen P. S. Garfinkel A. Qu Z. (2006). From pulsus to pulseless: the saga of cardiac alternans. *Circ. Res.* 98 1244–1253. 10.1161/01.RES.0000224540.97431.f0 16728670

[B45] Wilson L. D. Jeyaraj D. Wan X. Hoeker G. S. Said T. H. Gittinger M. (2009). Heart failure enhances susceptibility to arrhythmogenic cardiac alternans. *Heart Rhythm* 6 251–259. 10.1016/j.hrthm.2008.11.008 19187920 PMC2764250

[B46] Wilson L. D. Rosenbaum D. S. (2007). Mechanisms of arrythmogenic cardiac alternans. *Europace* 9(Suppl. 6), 77–82. 10.1093/europace/eum210 17959697

[B47] Xie L.-H. Sato D. Garfinkel A. Qu Z. Weiss J. N. (2008). Intracellular Ca alternans: coordinated regulation by sarcoplasmic reticulum release, uptake, and leak. *Biophys. J.* 95 3100–3110. 10.1529/biophysj.108.130955 18539635 PMC2527258

[B48] Yamamoto T. Yano M. Kohno M. Hisaoka T. Ono K. Tanigawa T. (1999). Abnormal Ca^2+^ release from cardiac sarcoplasmic reticulum in tachycardia-induced heart failure. *Cardiovasc. Res.* 44 146–155. 10.1016/S0008-6363(99)00200-X 10615398

[B49] Zhong X. Vallmitjana A. Sun B. Xiao Z. Guo W. Wei J. (2018). Reduced expression of cardiac ryanodine receptor protects against stress-induced ventricular tachyarrhythmia, but increases the susceptibility to cardiac alternans. *Biochem. J.* 475 169–183. 10.1042/BCJ20170631 29170159

[B50] Zhou X. Bueno-Orovio A. Orini M. Hanson B. Hayward M. Taggart P. (2016). In vivo and in silico investigation into mechanisms of frequency dependence of repolarization alternans in human ventricular cardiomyocytes. *Circ. Res.* 118 266–278. 10.1161/CIRCRESAHA.115.307836 26602864 PMC4719495

